# Ex Ante Knowledge for Infectious Disease Outbreaks: Introducing the Organizational Network Governance Approach

**DOI:** 10.1007/978-3-030-47150-7_14

**Published:** 2020-04-24

**Authors:** Jörg Raab, Patrick Kenis, Marleen Kraaij-Dirkzwager, Aura Timen

**Affiliations:** 2 Department of Geography, Heidelberg University, Heidelberg, Germany; 3 Department of Political Science, University of Chicago, Chicago, IL USA; 4 Department of Geography, Heidelberg University, Heidelberg, Germany; 5 Department of Organization Studies, Tilburg University, Tilburg, The Netherlands; 6 Tilburg School of Economics and Management, Tilburg University, Tilburg, The Netherlands; 7 Department for Environmental Health, Aftercare and Security (VLH), Dutch National Institute for Public Health and the Environment (RIVM), Bilthoven, The Netherlands; 8 National Coordination Centre for Outbreak Management (LCI), Dutch National Institute for Public Health and the Environment (RIVM), Bilthoven, The Netherlands

**Keywords:** Outbreak, Infectious diseases, Response, Governance, Stakeholders, Network, Transmission route

## Abstract

In our paper we investigate to what extent ex ante knowledge about a response network can be made available in order to deal with a crisis such as an infectious disease outbreak. Outbreaks are almost by definition characterized by a lack of information and knowledge. We introduce the organizational network governance approach for producing information prior to an actual outbreak, which is useful in limiting a virus’s transmission and impact. By introducing two fictitious but realistic outbreak scenarios—the outbreak of the West Nile Virus (WNV) and the outbreak of a New Asian Coronavirus (NAC) in the Netherlands—we demonstrate the effectiveness of this approach. We formulate recommendations how to use the generated information to strengthen the organizational context in order to limit a virus’s transmission and impact and how to further develop the organizational network governance approach. We also formulate recommendations for how to further develop the organizational network governance approach.

## The Importance of Knowledge in Infectious Disease Outbreaks

The world is regularly and increasingly confronted with the outbreaks of infectious diseases (Smith et al., 2014). In the Netherlands, for example, infectious diseases are a clear health risk (Volksgezondheidenzorg.info, n.d.). In 2010, more than 30,000 hospital admissions were related to infectious diseases and almost 18,000 patients were treated in outpatient care facilities. Despite structural control measures (e.g., immunization programs, clean water, hygiene, and sanitation efforts), new infectious diseases emerge due to factors such as increased trade through globalization, migration, and adaptation of microorganisms and can become serious public health issues. In addition to the morbidity and mortality of infectious diseases, outbreaks cause societal distress and large societal costs.

The large quantity of unknown factors makes it impossible to assess such an occurrence’s timing, duration, or path of development in advance. Given the uncertainty and complexity, the question is to what extent and how policymakers can prepare to control a large—possibly cross-border—infectious disease outbreak. In the present paper, we take up this task by advancing an approach to gain relevant knowledge to control the outbreak before it has taken place. This is an exceptional challenge for at least two reasons. First, outbreaks of infectious diseases very often show high complexity in the sense that they are characterized by incomplete, contradictory, and changing requirements that are often difficult to recognize and change during their development. Second, a large number of actors are likely to become active in case of an outbreak of infectious diseases, because they believe they have a stake in the outbreak. Other relevant actors, however, might not become active even though others think they should have a stake in the outbreak management. Consequently, an orderly response is unlikely and an unclear and even confusing set of actors will probably form. Improving capacity to respond to outbreaks of infectious diseases therefore requires researchers to gain knowledge on the evolving actor set and its interdependencies.

Asserting that knowledge is simply unavailable—given the wicked conditions mentioned above—is not an option considering what is at stake in controlling infectious disease threats (just think of the 11,000 persons who died because of an Ebola infection; Medaglini, Santoro, & Siegrist, 2018; Munjita, Chileshe, & Mutemwa, 2015). Consequently, in the present paper we develop an approach to the production of useful knowledge for preparing the control of infectious disease outbreaks. We label it the *organizational* *network governance* *approach,* which we build on three main arguments: First, the response to infectious disease outbreaks can best be conceptualized as an *organizational network response*; second, we can describe and analyze the structure of an organizational network using *network analytical methods;* and third, we can assess these networks in terms of their effectiveness in controlling disease outbreaks using *governance concepts*. We will demonstrate our approach by introducing two infectious disease threat scenarios and show the value of conceptualizing them as crisis-response networks, analyzing them as social networks and assessing them from a governance perspective. In this way, we create available knowledge for an effective response to infectious disease outbreaks.

## The Research Context

*Two fictitious but realistic scenarios of infectious disease* *outbreaks in the Netherlands*

This study is based on two fictitious but realistic outbreak scenarios in the Netherlands: an outbreak of a *New Asian Corona Virus (NAC)* and an outbreak of the *West Nile Virus* *(WNV)*. The *New Asian Corona Virus* is a fictive coronavirus but falls in the category of viruses emanating from Asia causing serious respiratory illnesses such as SARS (severe acute respiratory syndrome) or MERS (Middle East respiratory syndrome). There are no vaccinations or other preventative medicines to date. The virus is transmissible between humans via airborne infection or direct contact. There is no evidence for transmission via feces. Coronaviruses can cause various diseases in animals as well as people, varying from the common cold to severe respiratory syndromes. In humans, coronaviruses cause about 15–20% of upper respiratory infections. Disease symptoms vary by virus type, but coronaviruses often produce symptoms such as fever, coughing, fatigue, shortness of breath, and gastrointestinal symptoms like diarrhea. Some coronaviruses cause very serious symptoms, such as pneumonia, acute respiratory distress syndrome (ARDS), and multiorgan failure.

Much remains unknown about coronaviruses because they are hard to isolate. The first detection of an animal coronavirus in the laboratory was in 1937. Human coronaviruses were discovered in 1960. An outbreak of SARS-CoV (severe acute respiratory syndrome-related coronavirus) occurred in 2003–2004, causing over 8000 reported patients worldwide, of which about 10% died. In June 2012, scientists identified the first case of MERS-CoV infection in Saudi Arabia. The number of infected persons has exceeded 1800 cases globally, of which 640 have died. National and European systems are in place to notify and monitor important cases of MERS-CoV. In the Netherlands, MERS-CoV occurred in 2014 in two persons who travelled back from Saudi-Arabia.

The West Nile Virus is an arbovirus, which is transmitted from animals to humans or horses via mosquitoes (Bellini, Zeller, & van Borte, 2014; Weaver & Reisen, 2010). Wild birds are the primary enzootic cycle of WNV, with mosquitoes transmitting the virus amongst these wild birds (Bellini et al., 2014). When (climate) conditions permit, virus circulation may increase and spillover transmission via mosquitoes to humans or horses can occur. When transmission occurs, humans and horses usually serve as a dead-end host, meaning that not enough viral load is built up to infect mosquitoes. However, human-to-human transmission is possible following blood or organ donation from an infected donor. Although no symptoms occur in most human infections, in 20–30% of infections symptoms such as sudden onset of fever, headache, fatigue, and myalgias arise (Lim, Koraka, Osterhaus, & Martina, 2011), as well as gastrointestinal complaints with the risk of dehydration.

WNV can affect all ages, with high incidences among younger individuals, and among the elderly and immunocompromised. In addition, both susceptibility and the severity of the infection increase with age (Lim et al., 2011). Elderly people are therefore at higher risk of developing neuroinvasive disease, which may result in encephalitis, meningitis, or a poliomyelitis-like syndrome (Sejvar, 2014). Such outcomes are seen in less than 1% of infections, but are significantly more debilitating and lead to long-term outcomes in over 50% of cases (Lim et al., 2011; Sejvar, 2014).

There is no available treatment for WNV in humans, other than supportive care (Sejvar, 2014), which highlights the impact the disease’s introduction may have on a country (Rizzoli et al., 2015). For horses, on the other hand, vaccines are available to protect them from developing West Nile Fever and other WNV-related outcomes (Bowen et al., 2014; Iyer & Kousoulas, 2013).

National and European systems are in place to notify and monitor cases/the epidemiology of WNV (ECDC, 2013). A vast majority of European countries have reported either human or animal cases of WNV in the past, for example Greece, 2010 (ECDC, 2010); Turkey, 2010–2011 (Kalaycioglu et al., 2012); Croatia, 2012 (Pem-Novosel et al., 2014); Italy, 2012 (Barzon et al., 2012). To date, the Netherlands has had no autochthonous infections of WNV (i.e., infections acquired within the country) (Chancey, Grinev, Volkova, & Rios, 2014).

The two fictitious outbreaks would call for immediate responses or what is called outbreak management. Outbreak management is partially context specific, as control measures are related to the pathogen involved (characteristics of the virus or bacterium), the route of transmission (through inhalation, direct contact, sexual contact, oral intake), and the risk groups (related to many factors: age, immune response, and—very importantly—specific behavior). Risk groups can be those who become more easily infected due to exposure, or groups at larger risk of developing complications after an infection. It takes expertise to recognize an outbreak (understand the epidemiology and determine the source of infection, the mode of transmission, and the risk groups) and to develop effective and timely control measures.

In the Netherlands, it is the *Public* *Health Act* which regulates the response to events threatening public health in the Netherlands, including outbreak management (Wet publieke gezondheid, 2017). The National Coordination Center Communicable Disease Control (in Dutch: *Landelijke Coordinatie Infectieziektebestrijding*, LCI), a department of the National Institute for Public Health and the Environment (in Dutch: *Rijksinstituut voor Volksgezondheid en Milieu,* RIVM) (later labeled the National Coordination Authority ), is charged with coordinating actors within the response system if an outbreak involving different Dutch regions occurs (RIVM, 2019). The coordination of relevant actors is necessary in order to control the risks associated with an outbreak as effectively (less morbidity, mortality, and societal unrest) and efficiently (efficient use of human and financial resources) as possible.

To facilitate the formulation and implementation of control measures at the population level, an infrastructure for analysis and decision making is established (RIVM, 2012). The director of the Centre for Infectious Disease Control can invite the members of the Outbreak Management Team (OMT) to convene. The OMT is formed by a group of “fixed” experts, invited based on their personal expertise (e.g., communicable disease specialists, infectiologist, microbiologist, epidemiology, general practice; in case of a zoonotic disease, veterinary partners attend). The OMT is expanded based on pathogen- or context-specific needs (e.g., specific knowledge about risk groups, including specific veterinary expertise). The OMT advises the Board of Administrative Executives (in Dutch: *Bestuurlijk Afstemmings Overleg*), directed by the Director-General of the Ministry of Health. The BAO advises the Minister of Health on legal, financial, and political aspects of the proposed control measures. The minister of Health will interact with other ministers if collective control measures have an effect on, for example, trade, schools, or airports. Once the decision on collective control measures has been taken, the Minister of Health requests that the National Coordinating Authority to support actors in implementing the control measures with information and coordination as needed for an effective response.

Although there is a clear response system in place, it is evident that given the potentially broad societal and economic impact of the described scenarios, a myriad of actors within and outside the public health field will become involved.

The infectious disease outbreaks described are characterized by complexity in the sense that they entail incomplete, contradictory, and shifting requirements that are often difficult to recognize and change during their development. Moreover, numerous organizations, agencies, and other actors are likely to be involved in significant ways. Consequently, response patterns are emergent rather than routine or planned (Majchrzak, Jarvenpaa, & Hollingshead, 2007).

## The Research Challenge and Theoretical Approach

Given the empirics of the diseases described above, actors search for relevant knowledge to control an outbreak before it takes place. More specifically, we concentrate on the question whether investigating the multiplicity of actors related to a crisis as well as the relational pattern of knowledge seeking and sharing between these actors provides a useful knowledge base for controlling outbreaks of infectious diseases (Borgatti, Everett, & Johnson, 2018). The questions become: Who are the actors related to an outbreak of a specific disease, what are the patterns of relationships between these actors, and what information do these possess that are relevant for controlling an infectious disease outbreak? Before presenting our findings based on an analysis of two fictitious disease outbreaks, we discuss our research’s theoretical foundation.

We propose conceptualizing a situation of an infectious disease outbreak as an *organizational* *network response*, in which a myriad of actors will become active while others stay inactive (nevertheless others expecting them to become active). Different types of relationships will or will not develop between these actors, resulting in a system of information sharing, command, collaboration , and so forth (Glückler & Panitz, 2016). The assumption is that this so-called organizational network decisively influences the response’s development and quality. We base such analysis of crisis response from an *organizational network-response* perspective on the following assumptions. First, we begin our organizational network-response from a realist perspective and do not a priori include or exclude certain organizations that should (or should not) be part of the response. We thus widen our lens to include possible peculiarities of the crisis leading to improper measurement or missing important knowledge (see, e.g., Weick’s (2006) study on the WNV incidence in new NYC in 1999). Indeed, stakeholder analysis has become important in crisis response analysis, but few studies exist whose researchers examine crisis response from an overall network perspective (compared to ego-centric perspectives, with stakeholders as alters). Second, such an approach opens a perspective for studying the network positions and interactions between the different actors in the networks, thus producing knowledge about the network’s dispersion, information flow, leading organizations, the presence of peripheral groups, and so on (Glückler & Doreian, 2016). Third, such an approach serves as a basis for better understanding how collaboration and communication between actors can be improved (Moon et al., 2015; Swaan et al., 2017; Vinck et al., 2011), linking its findings to a governance perspective. We here define governance as the structures and interactive processes that steer actors’ activities towards a common goal (Ansell & Torfing, 2016; Kenis, 2016). Whereas networks describe the actors and the relational patterns between these actors, a governance perspective adds the question of whether and how these networks lead to network outcomes. Given the absence of market logic or classical hierarchical logic, the question becomes which mechanisms steer the network’s functioning. We consider these mechanisms essential to understanding how a set of organizations and its relational patterns function in a network form of organization. We here define actors, relational patterns, and mechanisms as network-level governance (Glückler, Dehning, Janneck, & Armbrüster, 2012).

The organizational network governance approach proposed here is first and foremost an analytical one, whose utilizers attempt to create knowledge to improve response preparedness by conceptualizing the response system as a network based on actors and their ties. Network is thus used as an *empirical tool* (Raab & Kenis, 2009). As stated above, however, we believe that in a situation where neither market nor hierarchical mechanisms seem likely to work, as is the case for international infectious disease threats, network as a form of governance or *governance tool* is the most likely and appropriate option. Whether the most appropriate response is based on a shared governance mode, lead organization or network administrative organization (Provan & Kenis, 2008), or a mixed form (Berthod, Grothe-Hammer, Müller-Seitz, Raab, & Sydow, 2016) is an empirical question and depends on several factors, of which the formal legal framework in which the response takes place is likely an important one.

The organizational-network response approach resonates with recent observations in the field of crisis management. Recent research on organizational networks in general and their use as a tool to respond to disasters and emergencies has significantly improved our understanding of the structure, governance, functioning, and effectiveness of such systems. In addition, the field of public sector networks in general has made important progress regarding the governance of goal-directed networks (Raab, van den Oord, & Kenis, 2015). Provan and Kenis (2008), for example, have provided the field with a conceptual vocabulary and specific lens that has helped researchers to better analyze the different forms of network coordination in general (Ansell & Gash, 2008; Emerson & Nabatchi, 2015; Glückler, Lazega, & Hammer, 2017; Provan, Fish, & Sydow, 2007; Raab, Mannak, & Cambré, 2015) and emergency response systems in particular (Berthod et al., 2016; Moynihan, 2009). In addition, the 9/11 terror attacks in New York and Washington triggered a whole stream of research on interorganizational response networks (Hu, Knox, & Kapucu, 2014; Kapucu, 2009; Moynihan, 2009; Nowell & Steelmann, 2015). The perspective of an organizational network-response was recently also proposed in a report on “New Directions in Governing the Global Health Domain” (Kickbusch, Cassels, & Liu, 2016). Its authors concluded that those dealing with health challenges will in the future need to widen their lens to include actors who lie outside what has traditionally been defined as the infectious disease architecture. This is due to considerable failures in responses (e.g., Ebola; Moon et al., 2015; Stoto & Higdon, 2015) and the increasing interdependencies in today’s world. Public health specialists in the Netherlands have also recognized this situation (Huizer, Kraaij-Dirkzwager, Timen, Schuitmaker, van Steenbergen, 2014; Kraaij-Dirkzwager, Schol, Schuitmaker-Warnaar, Timen, & van Steenbergen, 2019).

How, then, can one usefully describe and analyze an organizational network-response that results in reaction to a disease outbreak? Given the fact that we conceptualize the situation as one with an a-priori unknown set of actors and among which the interaction has an important effect on controlling an outbreak as early as possible and with minimal consequences, we propose network analytical tools as an appropriate approach. The use of such tools goes beyond the more common mapping of the relevant actors and providing a generic list of all possible actors involved and will be introduced in the following.

We thus explore the potential governance system for these two fictitious but realistic infectious disease outbreaks through a network lens. Knowledge that we will acquire through this exploration will likely help in fighting future outbreaks in the following way:

1. Which actors are mobilized? Are these all the appropriate actors? Some actors are frequently not mobilized because they are not part of the core actor set within public health, but are nonetheless crucial for outbreak management.
2. To what extent are those that are deemed important willing to engage? Ideally, we would like to see actors on the diagonal, in other words, the more important, the more engaged.
3. In terms of network structure, how are the relevant actors connected?
  If actors form clusters and these are connected by brokers, these brokers should have the competence and capacity to function in such a connector role.
4. In terms of governance, to what extent are core health care actors well and densely connected in the center, collectively coordinating the response under the LCI’s leadership and well connected (but more sparsely) to the more peripheral actors?

Public health authorities can utilize these insights to develop a relational lens with which to analyze, structure, and manage the emerging organizational network response.

## Introducing Network Analytical Tools for Studying Infectious Diseases Responses

Network analytical tools are useful for describing the (in)formal relationships in an evolving crisis-response network. Insight into the outbreak networks described in the scenarios above provides parallel insights into the unfolding communication and coordination at the level of the crisis-response network.

Network analysis is a method of collecting, analyzing, and visualizing relational structures and processes. The network perspective is now more than a metaphor: It is a systemic way for researchers to study how societies with their individuals, communities, and organizations interrelate, based on and with its own theoretical statements, methods, and research findings (see Borgatti et al., 2018; Rainie & Wellman, 2012, for an overview). Proponents study and describe systems of interaction in terms of both their actors (called “nodes” or “vertices” in the language of network theory) and relationships (called “links”, “ties” or “edges”). Links can be of many types, such as information exchange, trust, exchange of resources, and the like. Networks are usually represented by data matrices and related diagrams, in which the units are represented by points and lines between them, either with or without arrowheads dependent on whether they have a direction or not.

Network analysis researchers have developed a great and powerful number of ways to describe networks characteristics, which we have summarized in Table 14.1.

**Table 14.1:** Overview of basic network descriptors

Dimension	Indicator	Rationale for use in empirical analysis
Derived attributes of actors	Centrality	Indicators of centrality identify the most important node (actors) within a network. Given the fact that “important” can mean different things (depending on the research questions) there are also different centrality indicators. For example we can look at the “importance” of an actor (e.g., who is closest to all other actors) or the importance of the actor in the cohesiveness of the network. The most commonly used centrality indicators are: degree, closeness and betweeness. Eigenvector centrality assigns relative scores to all nodes in the network based on the concept that connections to high-scoring nodes contribute more to the score of the node in question than equal connections to low-scoring nodes.
	Status, prestige, prominence	In a directed network, a tie is not a symmetric connection between two actors, but an asymmetric link, going from one actor to another leading to difference in prominence or prestige among actors. The simplest measure of prominence for directed networks simply breaks down the degree count for incoming ties (in-degree) and outgoing ties (out-degree). Many other centrality measures can be similarly adapted to directed networks and used as prestige measures as well.
Structural roles of actors	Bridge, broker, …	Apart from the absolute number of ties an actor in a network can have specific positions in a network. An actor who is connected to actors who are themselves not directly connected has opportunities to mediate between them and the actor itself or the overall network can profit from this mediation.
Structural partitions	Communities	A network is said to have a community structure if the nodes of the network can be easily grouped into (potentially overlapping) sets of nodes such that each set of nodes is densely connected internally. The inhomogeneity of connections suggests that the network has certain divisions within it. The most commonly used indicators for analyzing community structures in networks are: Clique analysis, cluster analysis, and block analysis.
Derived network attributes	Density	Density indicates the general level of cohesion in a network. It calculates the proportion of direct ties in a network relative to the total number possible. Denser networks are not by definition better than sparser networks. This is contingent on the research question and perspective. Would we like to see ‘old boys networks’ to be more dense or less dense?
	Centralization	Centralization refers to the overall cohesion or integration of a network. Networks may, for example, be more or less centralized around particular actors or groups of actors. Most centralized for instance is a star network.
	Cohesiveness	It refers to the minimal number of actors in a social network that need to be removed to disconnect the group. This might be important for understanding how social networks shape communities, facilitate norm maintenance, or form the basis of categorical group identity, among many other things.
	Connectedness	The inventory of the total connections among actors is useful for getting a sense of how closely coupled the entire network is. This information can be used to understand how information moves in the network.
	Subgraphs and components	This indicates that the graph can be partitioned in certain ways. A *subgraph* is a subset of the nodes of a network, and all of the edges linking these nodes. Any group of nodes can form a subgraph. Components, on the other hand, are portions of the network that are disconnected from each other.

*Note.* Reprinted from “Analyzing policy-making II: Policy network analysis”, by P. Kenis and V. Schneider, 2018, in M. Puppis, K. Donders, L. van Audenhove, & H. van den Bulck (Eds.), *The Palgrave handbook of methods for media policy* *research* (pp. 471–491), Cham: Palgrave Macmillan. Copyright 2018 by Springer Nature. Reprinted with permission

## Measures, Data Collection, and Data Analysis

We have operationalized the data collected for the present study in order to contribute to the knowledge required to help fight future outbreaks in the following ways:

### The List of Actors in the Crisis-Response Networks

In order to mirror reality as best as possible, we used two fictive scenarios as the basis for our network analyses. One scenario described the early onset of a West-Nile Virus (WNV) outbreak with several autochthonous cases among humans and horses in the Netherlands. The second scenario described a rapidly evolving outbreak of a new coronavirus (NAC) after introduction through a traveller returning from Asia. (Full descriptions of the cases are available on request.)

Because the networks in both cases included actors from inside and outside the public health field, we used exploratory interviews and two focus groups with infectious disease control experts (n = 6 and n = 7, respectively) to determine the network boundaries and to develop the questionnaire. In addition, we had to find a way to combine concrete organizational actors like the National Coordination Authority with actor groups like general practitioners, boards of academic hospitals, emergency physicians, microbiologists, infectiologists, or veterinarians. With the help of the two focus groups we defined a relevant actor as: “any organization and/or representative that has a positive or negative influence on the prevention, control, treatment, and/or decision making with regard to (the outbreak of) the infectious disease at hand.” We also attributed three main characteristics to actors that influence their role during an outbreak: their level of influence on outbreak management (related to their interest in acting and potentially contributing), the amount and type of knowledge and information they have available (related to their position in the network), and the level of collaboration they engage in. We asked respondents to focus on the potential collaborations and coordination with other relevant actors in the Netherlands as the scenarios allowed. Based on the generic list of actors available at the National Coordination for Communicable Disease control, the focus groups identified 98 possibly relevant actors for the WNV scenario and 61 potential actors in the NAC scenario. We thus applied a realist strategy in determining the network boundaries.

We did not include nonorganized stakeholders such as infected individuals, travellers, recreational water/land users, hunters, farmers, and gardeners (i.e., specific risk-groups) or professionals who were already represented through other organizations (e.g., equine specialists through the Animal Health Services and Royal Netherlands Veterinary Association). To include a representative sample of all these stakeholders would mean a disproportional effort at this stage of our exploratory research. We excluded media for the same reason. We approached some stakeholders via an umbrella organization—those we judged to be extremely relevant but difficult to access directly—for example: boards of the academic hospitals (via the Netherlands Federation of University Medical Centres (NFU)) or microbiologists (via the Dutch Association of Medical Microbiology (NVMM)). By asking the associations to select five representatives from different regions to fill in the questionnaire, we argue that a proper reflection of their information flows would be captured without burdening too many people. In addition, in the case of two organizations—with formal tasks related to infectious disease control and disaster/ crisis management in the health care sector—that are located throughout the Netherlands, all divisions were approached to obtain insights into potential regional differences within institutes (i.e., Municipal Health Services (GGD) and Regional Consultation on Acute Care (ROAZ)).

### Data Collection and Types of Ties

We piloted the questionnaire and adapted it accordingly (questionnaire available on request in Dutch). We first asked respondents to indicate their role(s) and potential contributions (unique skills and capabilities) to outbreak control, and then introduced the scenarios. Second, we incorporated a list of control activities (approved by the focus groups of control experts) and asked respondents to indicate in which control activities they were likely to be involved. Third, the questionnaire contained a table in which we asked respondents to indicate per organization identified if they would obtain and/or provide information/advice from and to this organization. Fourth, we asked the respondents to indicate with whom of the stakeholders they would expect to have the most intense collaboration , plus their expectations of the activities the particular stakeholder would undertake. Finally, the respondents were asked to indicate their perceived level of influence over the outbreak control in the scenario provided and their level of interest in being involved in outbreak control.

We therefore based the network analysis on three types of ties: joint involvement in control activities, providing and receiving information/advice, and collaboration . For actor groups (health care professionals), we coded a tie as existent (=1) if at least 50% of respondents from an actor group—for example, 50% of the general practitioners—indicated a relationship, and did additional robustness checks, for example, 60% of respondents or at least one respondent.

We used Visone (Brandes & Wagner, 2004) to analyze and visualize the network data. We calculated general descriptive network measures for the actor level, such as degree, (flow) betweenness, closeness, and status. We further used the spring embedder algorithm implemented in Visone to identify possible clusters and visualize the two mode networks of actors and measures for outbreak control in order to analyze how the response measures and actors are connected and between which actors coordination would be required. For joint involvement we used a spring embedder analysis to identify clusters of actors around certain control measures. For information/advice provision/reception we applied flow betweenness centrality and status, and in case of collaboration we again used the spring embedder to identify cliques and brokers.

### Data Collection

The questionnaires were sent to the representatives of the actors or selected respondents from actor groups. A reminder was sent 10–14 days later. If a response still had not been received after 5 days, the organization was mailed or called as an extra reminder to minimize nonresponse. In total, in the NAC case we included 43 actors or actor groups in the data analysis, which represents a response rate of 80%. For the WNV case, we included 82 actors or actor groups in the data analysis, which represents a response rate of 82%.

### Data Analysis

We discussed the analyses’ preliminary outcomes during a 1h focus group with communicable disease control specialists (medical doctors, nurses, and policy officers); public health specialists (medical doctors and policy officers); microbiologists; entomologists; researchers with an interest in crisis management, preparedness, and response related to infectious disease threats; and guideline developers.

## Results

In the following we present the results with actors in an anonymized form (confidentiality was promised to the respondents). We present the actors who have been named relevant in the two settings, their involvement, and the distribution of information.

### Actors in the Two Networks

An important question in infectious disease outbreak response is, of course, which actors should be involved to manage the outbreak. This is a tricky issue. Compared to simple or even complicated tasks in which one could specify in advance what the perfect task division would be to get the task done, this is quite different when we are dealing with “wicked problems” (Head, 2008) or those as complex as explained above. Consequently, the first question researchers using an organizational network response approach ask is: Who is or should be part of the response system? Often, actors who are not part of the core of the public health system might need to be involved as early as possible to limit the impact of the outbreak.

The larger number of actors in in the WNV scenario is due to the fact that transmission happens via mosquitos and animals and therefore a large group of actors becomes relevant that does not belong to the traditional (human) public health field such as hunters, veterinarians, water management associations, or the Ministry that deals with agricultural and nature issues in the Netherlands. Different actors constitute the core of the response network in the NAC and the WNV scenario. For instance, an organization with the mandate to control mosquitoes and coordinate specific veterinary measures are prominent in the core of the network controlling the WNV outbreak.

### Actor Involvement in the Two Networks

**Fig. 14.1:**
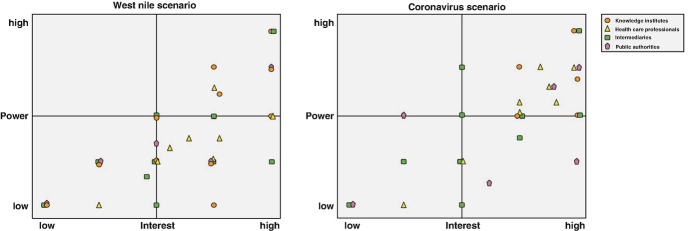
Power interest matrix for the two scenarios. Source: Design by authors

Here we focus on to what extent the actors have an interest in actively participating in the impact of the outbreak. This is an indicator for whether they see a role for themselves in the organizational network response. In order to answer this question, we created a power-interest matrix (see Fig. 14.1 above). A power-interest matrix combines two types of information: whether the actors see a role for themselves in the response and whether they are interested in playing that role. The first tells us something about the task allocation and the second tells us something about how rewarding they consider their participation or, in other words, how interested they are in becoming active in the response. The crosstabulation of these two dimensions proves particularly helpful, because it can point to the discrepancy or tension between the necessary task allocation and the degree of interest in performing one’s task.

In these figures, we show different types of actors (knowledge institutes, health care professionals, intermediaries, and public authorities) and their specific position in the power interest matrix. Some points in the matrix represent more than one actor of the same type. Actors are willing to indicate their self-perceived interest and power and differentiate their role in the scenarios (e.g., health care providers estimate a larger role for themselves in the NAC scenario than in the WNV scenario). For coordinating parties in the response system, this provides an interesting mirror for their own expectations of actor involvement, a starting point for dialogues with actors/groups about possible fulfillment of their role in a specific context.

The power-interest matrix (above) also provides an interesting mirror for the actors’ own expectations of their involvement and a starting point for dialogues with actor(group)s, including about possible rewards and incentives to fulfill their role in a specific case and context.

In both scenarios, we see a general linear relationship between self-perceived power and interest, with the north-west quadrant remaining empty. This signals that many actors who think they have the most power to intervene in the transmission also indicate a strong interest in participating. Interestingly, there are also actors with self-perceived low power who (might) have crucial skills, assets, or capacities at their disposal. The actors in the northeastern quadrant can be described as the core actors in the response system in their self-description with high power and high interest. It is interesting, for example, that an organization that engages in extermination of mosquitos is very much aware of their potential role and importance in the WNV scenario, and that the health care professionals (yellow triangles) voice strong interest in both scenarios but assess their power to be much less in the WNV scenario (most health care professionals are located in the southeastern quadrant in the WNV scenario, but appear in the northeastern quadrant in the NAC scenario).

**Fig. 14.2:**
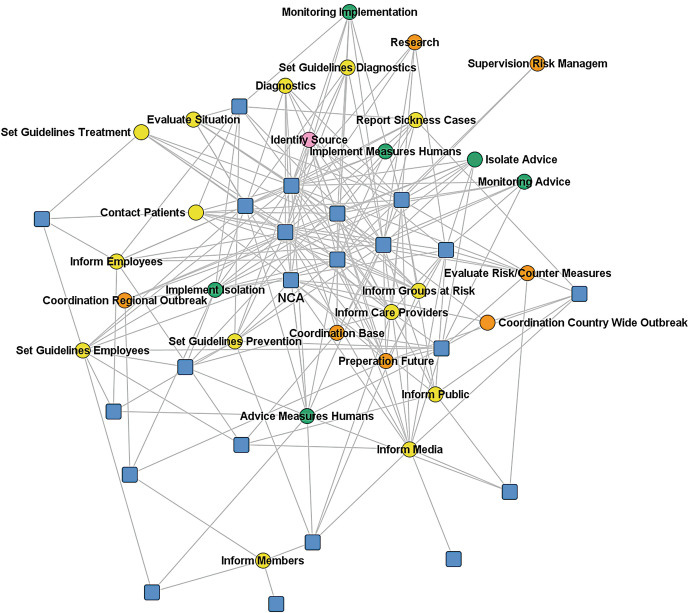
Two-mode network based on involvement in reactive measures (NAC). Source: Design by authors

**Fig. 14.3:**
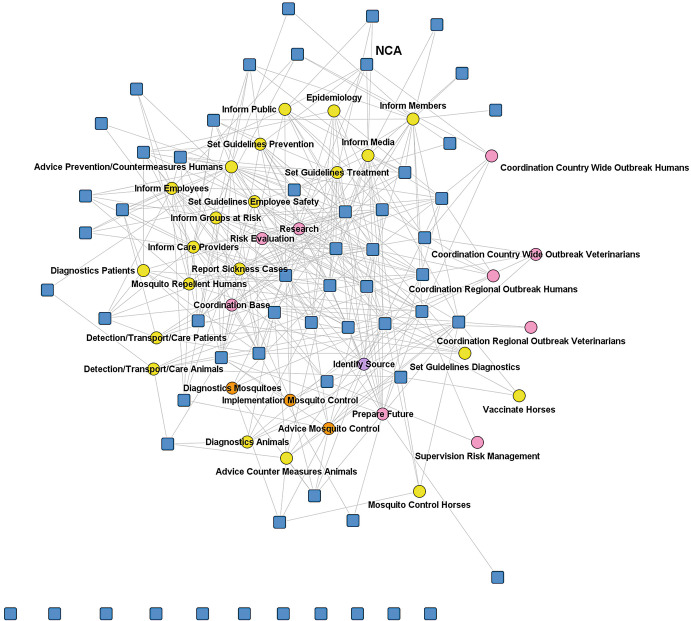
Two-mode network based on involvement in reactive measures (WNV). Source: Design by authors

Actors’ involvement regarding specific measures to fight and control the outbreak can also be seen as actors indicating the tasks they plan to perform in the outbreak scenarios. In the next two figures we depict the respondents’ answers to the question “in which of the following outbreak control measures is your organization involved in this scenario.” We had identified 28 measures in the NAC scenario and 34 in the WNV scenario as necessary for reacting to and controlling the outbreak, from which respondents could choose. Blue squares represent actors; circles the different measures, which were grouped in identification of the infection source (pink), developing guidelines and informing health care providers and risk groups (yellow), developing and implementing of control measures (green), coordination, evaluation, and research (orange). The measures clearly represent the operational level of reacting to and controlling an outbreak (Fig. 14.2 and Fig. 14.3).

For the NAC scenario, we can observe nine core actors in the middle who are involved in taking different measures and are therefore also likely or at least could potentially coordinate the application of these diverse measures. However, there is no clear clustering of measures and actors, which means that actors and measures of different types are closely connected and that there are no separate actors who deal exclusively with specific measures. Therefore, the integration of the different types of measures and the measures themselves seems quite high but not very structured. A disadvantage of this situation, therefore, could be that it requires significant conscious additional coordination, which is relatively inefficient if there is no formal structure with mandated or natural coordinating actors (other than the National Coordination Authority/NCA). Ideally, some actors would simultaneously be involved in certain measures, and others would be specialized and involved only in a limited number of other measures.

In the NAC scenario, these nine core actors represent some of the most important public health actors in fighting an outbreak, with other actors scattered more at the periphery. This analysis implies that when it comes to the coordination of measures, it is mainly these nine important public health actors that need to reconcile their strategies and actions on the operational level.

In the WNV scenario, the network is characterized more by a bifurcated structure with some actors on one side and the others on the other, connected by different measures regarding information delivery and (public) communication.

As far as the task allocation is concerned, therefore, many operational tasks are performed in the two organization network scenarios and similar tasks are often performed by several organizations. These initially look like rather uncoordinated systems, but to better understand the coordination of tasks in the systems we asked respondents to nominate up to five actors with whom they would work together most intensively in such a scenario regarding limiting transmission of the virus and its impact. This is a good indication of the division of labor which would evolve in the network.

**Fig. 14.4:**
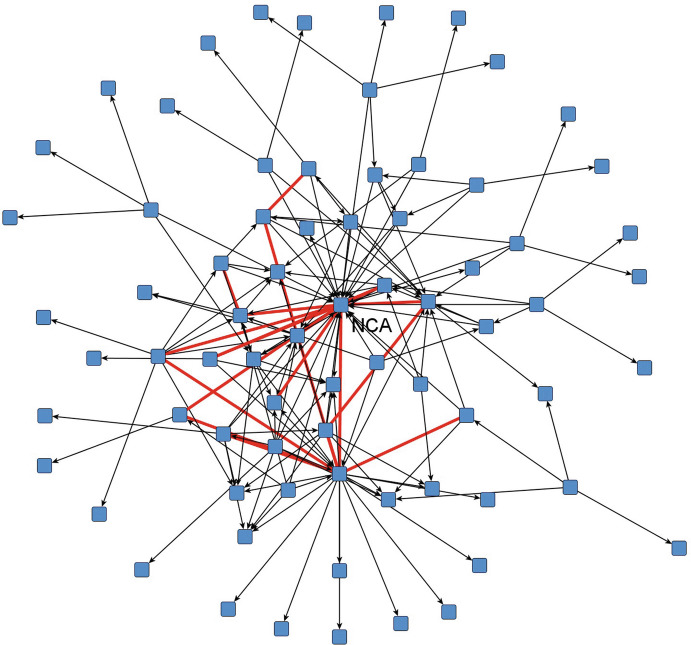
Collaboration between actors (NAC scenario, min 1x mentioned, red lines reciprocal). Source: Design by authors

**Fig. 14.5:**
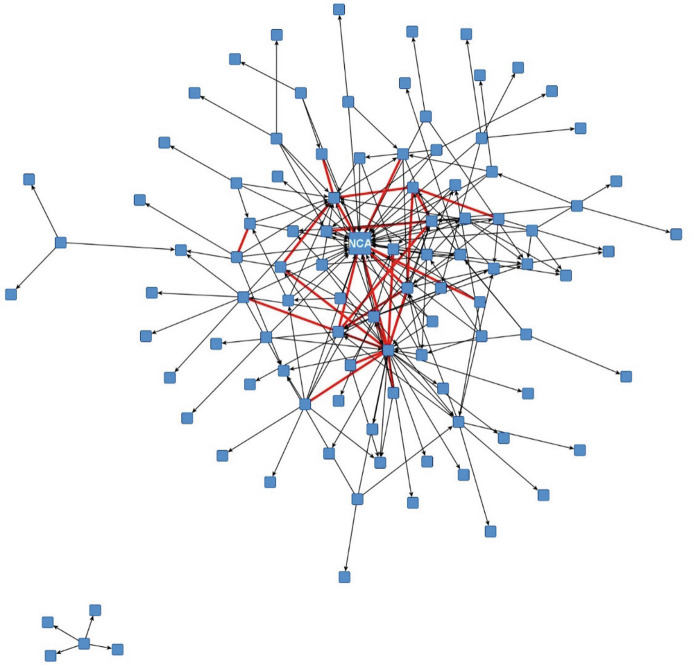
Collaboration between actors (WNV scenario, min 1x mentioned, red lines reciprocal). Source: Design by authors

In Fig. 14.4 and Fig. 14.5 below, we depict the structure based on the type of tie “collaboration” . The ties are directed to indicate the nomination. Red undirected relations (the thicker lines) indicate that both actors have nominated each other reciprocally. These ties, in other words, connect those groups/actors one would expect to have the most intense collaboration with respect to outbreak control in the specific scenario, and also indicate that organizations hold expectations towards each other about their respective roles and contributions in outbreak management.

We have visualized the network using a spring embedder algorithm, placing nodes in such a way that connected actors are attracted to each other and placed close to each other, and actors who are not connected repel each other and are placed further apart. Through this algorithm, network clusters become visible.

For both scenarios, one can observe that a relatively dense core exists where actors also frequently nominate each other in a reciprocal way. Due to the higher number of actors, there are also more collaboration relations in the WNV scenario. Interestingly, a lot of directed and thus unreciprocated nominations also exist at the network’s core. An interesting question to follow-up is, of course, what the ratio of these hubs is and the ratio of the connections between the hubs. Are these hubs and spokes in effect confirming the law of homophily, which would imply that similar actors are more likely to collaborate with each other? This situation is not necessarily the most appropriate one in a situation of complexity, where one would expect a more integrated approach to collaboration .

###  Information Distribution in the Crisis-Response Networks

 Information provision is about the information actors need to perform their task and coordinate their work with others. In what follows, we do not analyze the need, but the actual nomination of which actors provide information to other actors. The question becomes to what extent the information provision reported contributes to the goal of the organizational network, in turn limiting the transmission of the infectious disease and its impact.

**Fig. 14.6:**
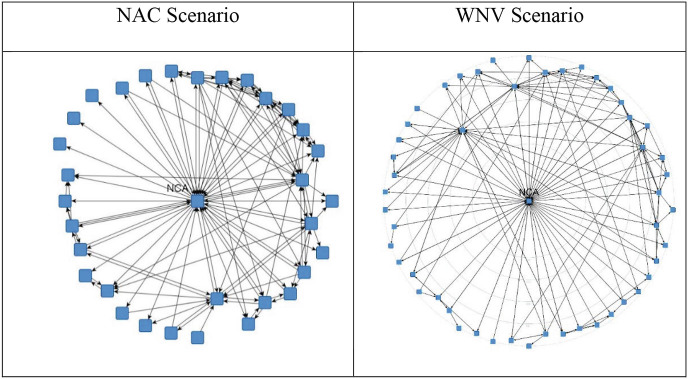
Flow Betweenness Centrality based on giving information/advice (confirmed ties). Source: Design by authors

In Fig. 14.6, we depict the network structure in a centrality layout with regard to the flow betweenness of the actors based on the confirmed ties “giving information/advice” in case of a virus outbreak. Actors are regarded as central if they lie on a path between any two other actors. Ties are seen as pipes through which information can flow. The more often an actor is on such a path, the more important it becomes for the transmission of information. We assume that information in principle travels on the shortest path between any two actors but that it might also travel on longer paths (though is less likely to do so). Therefore, the shorter the paths are, the more weight they receive in calculating the flow betweenness centrality.

In Fig. 14.6, we show that the communication network is relatively centralized, with the NCA in the most central position. Although a lot more actors are involved in the WNV scenario, the structures in the two scenarios very much resemble each other, with the Ministry of Health and the Municipal Health Agencies (actors with formally described roles in the Public Health Act) in the following ranks with some distance to the NCA. The centralization of the communication structure around the NCA implies a lead organization type network in terms of governance, because the NCA is operationally involved and clearly by far the most central actor. However, there are also many linkages between the other actors.

Although links between the alters are present and encouraged in the ideal typical model suggested by Provan and Kenis (2008) to avoid information overload in the center and foster innovation, one might wonder how such a considerable number of links impact network governance. We believe this may prove a particular challenge when it comes to formulating a consistent message to the public and to health care professionals.

One possible solution for information provision and coordination is a network that is characterized by a central entity with more decentral hubs or brokers. With such a network structure, information provision and coordination can be achieved relatively efficiently through local hubs that each are responsible for a certain cluster or section of the network. Researchers have reported on this type of solution in earlier studies (Lemaire & Provan, 2009; Moynihan, 2009).

**Fig. 14.7:**
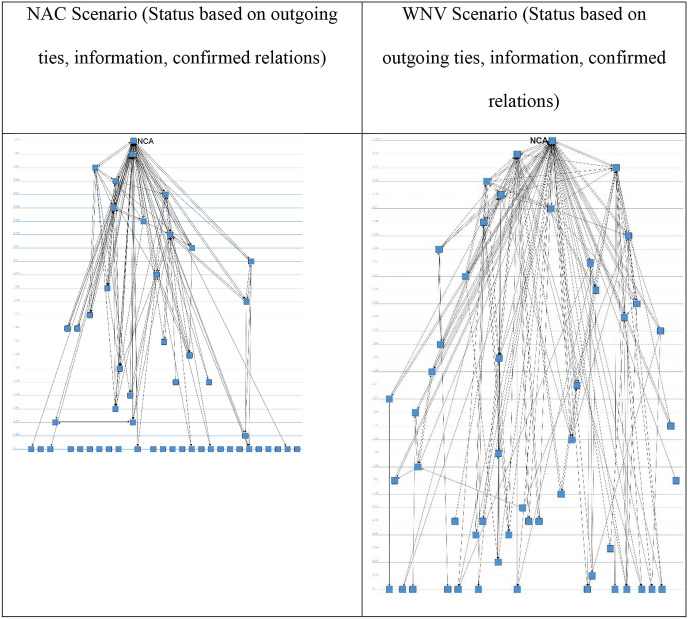

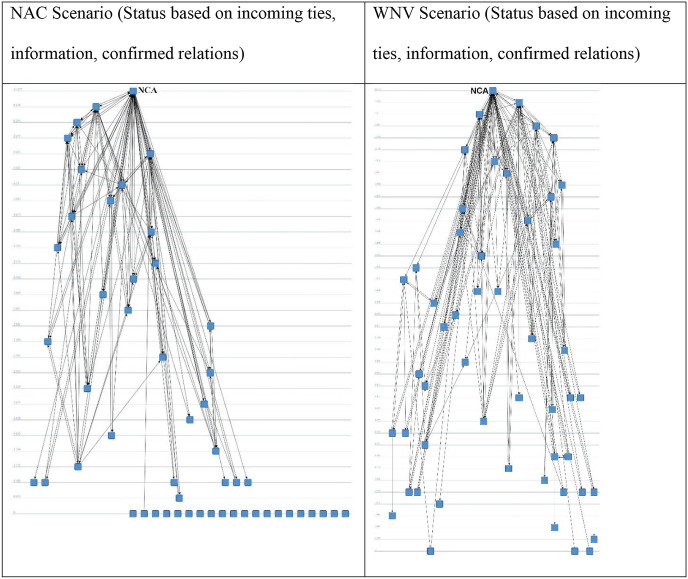
Actor status on the basis of confirmed information ties. Source: Design by authors

In Fig. 14.7 below, we depict the structure based on confirmed ties with regard to giving and receiving information or advice in terms of reacting to and controlling an infectious disease outbreak in the NAC and WNV scenarios. Compared to Fig. 14.6, the actors’ prominence is not determined through flow betweenness centrality but by their status. In the two visualizations in the upper row, we have calculated status on the basis of outgoing ties; in other words, the more information ties actors have to other actors who again spread information to many other actors, the more prominently they are positioned; in other words, these can be labeled as *super spreaders*, to use a term from diffusion theory. This is reversed for the visualizations in the second row. Here, actors are the more prominent the more information they receive from organizations that already receive a lot of information. In this way, top receivers of information can be detected.

In both cases, the NCA evidently holds the top position. A set of about 10–15 organizations also exists that forms the top of the information distribution pyramid. It becomes clear that some actors are both top information spreaders and information receivers, such as the NCA, the Ministry of Health, and the municipal health services. On the other hand, some actors are primarily receivers of information. The questions regarding the preparedness are then: Are the appropriate actors in the important positions, are the knowledge institutions well connected with the actors that make decisions and have coordination tasks, and are any actor positions in danger of information overload?

In Table 14.2, we summarize the results for the two scenarios.

**Table 14.2:** Summary of results

	NAC scenario	**WNV scenario**
# Actors	61	98
Type of actors	Mainly core public health actors	Public health actors but also hunters, veterinarians, water management associations or the Ministry of Economic Affairs that deals with farming issues
Actor engagement (power-interest matrix): Self assessment, how much interest an organization has in participating in a response and its perceived influence over the outbreak control	General linear relationship between self-perceived power and interest with the north-west quadrant being empty, in other words, no actors indicating high power, but low interest in outbreak management	General linear relationship between self-perceived power and interest with the north-west quadrant being empty, that is, no actors indicating high power, but low interest. Several actors, especially groups of health care professionals indicating high interest but only medium or even low power in outbreak management
Joint involvement (spring embedder, two mode): In which control activity will your organization be involved?	28 control measures identified Similar tasks are often performed by several organizations. 9 core actors of important public health actors that are involved in taking different measures and could potentially coordinate the application of these diverse measures. However, there is no clear clustering of measures and actors, which likely requires significant conscious additional coordination	34 control measures identified Similar tasks are often performed by several organizations. Bifurcated structure with some actors on one side and the others on the other connected by different measures regarding information delivery and (public) communication
Collaboration (spring embedder): With which of the organization do you expect your organization will have the most intense collaboration?	Relatively dense core exists where actors also frequently nominate each other in a reciprocal way. However, also a lot of asymmetric nominations	Relatively dense core exists where actors also frequently nominate each other in a reciprocal way. Due to the higher number of actors, there are also more collaboration relations in the WNV scenario. However, also a lot of asymmetric nominations
Information distribution: Provision/reception of information/advice (*flow betweenness*): To which of the organization will your organization provide information/advice, from which will it receive information/advice?	Structure is relatively centralized with the NCA in the most central position. Around the NCA implies a lead organization type network in terms of governance, since the NCA is operationally involved and clearly by far the most central actor. However, we also see a lot of linkages between the other actors	Structure is relatively centralized with the NCA in the most central position. Even though there are a lot more actors involved in the WNV scenario, the structures in the two scenarios very much resemble each other with the Ministry of Health and the Municipal Health Agencies (actors with formally described roles in the Public Health Act) in the following ranks with some distance to the NCA
Information distribution: Provision/reception of information/advice (*status*)	NCA holds the top position both in receiving and providing information. There is also a set of about 10–15 organizations which form the top of the information distribution pyramid. Analysis shows actors that are both top information spreaders and information receivers like NCA, the Ministry of Health, and the municipal health services. On the other hand, there are actors that are primarily receivers of information	Very similar structure compared to NAC scenario despite larger number of actors

*Note*. Source: Design by authors

## Discussion and Conclusion

In this paper, we present the organizational network governance approach as a way to analyze the response to a potential infectious disease outbreak. Although similar approaches have been gaining ground in the recent past to analyze organizational responses to disasters (Kapucu, 2009; Nowell, Steelman, Velez, & Yang, 2018) and to infectious disease outbreaks (Bdeir, Hossain, & Crawford, 2013) in particular, most researchers have conducted a retrospective analysis; in other words, they look at the response and the structures and governance that evolved after the fact. However, if the field wishes to improve preparedness for often uncertain threats, it must find a way to assess the capabilities and capacities of a response system to deal with a disaster or an outbreak before an incident happens. Researchers should therefore ideally gain information ex ante about the potential organizational network response to increase preparedness.

In this paper, we have developed a specific and feasible approach to produce such knowledge to limit transmission of the infectious disease and its impact. We have based this approach, on the one hand, on the observation that infectious disease threats by definition provoke an organizational network-response and, on the other hand, the fact that this response can fruitfully be analyzed using social network analysis. The fact that an infectious disease threat provokes an organizational network-response was confirmed by our data, which we collected for two fictitious but realistic scenarios of infectious disease outbreaks in the Netherlands, an outbreak of a New Asian Corona Virus (NAC) and an outbreak of the West Nile Virus (WNV). In both cases, respondents named a very high number of different organizations, with a substantial number of organizations outside the classical health architecture, as having a task in responding to the infectious disease threat.

Of course, such an ex ante approach with fictitious but realistic scenarios does not limit the need for, and usefulness of after-action assessments. Although collection of relational data might suffer from recall bias, especially if the incident is farther in the past, relational data based on scenarios might suffer from a social desirability bias. What one might do could differ from what would happen in reality. However, vignette studies, which are similar to our approach, have been proven to be a useful tool for research for some time and we have tried to minimize the potential bias in the relational data by basing the analysis on confirmed ties.

We continued our analysis by applying social network analysis to the scenarios of infectious disease outbreaks and arrived at several observations. We saw a large and highly differentiated network emerge in both cases. The network in the WNV scenario is much larger due to the different transmission paths (not human to human but via animals and mosquitoes). This results in the involvement of many more actors, especially those from outside the traditional (human) public health system, such as mosquito exterminators, water associations, and the ministry responsible for agriculture and nature. Attention needs to be paid to those actors that presumably have a strong influence but little interest. On the other hand, we can also identify some actors that assess themselves as having low power, although they appear quite important in the status analysis. The information structure is relatively centralized around the National Coordination Authority (NCA), which would imply a lead organization structure. However, in the analysis of the actor-measures structure, we see other actors besides the NCA as potential coordinating actors. If the NCA had an exclusive coordinating role, one would expect the NCA to serve as an exclusive connector between central measures to control the outbreak. Both networks are well integrated but not in a very structured way, which might lead to inefficiencies, difficulties creating a common information policy , and a risk of overburdening certain actors while not meeting the specific ( information) needs of other actors. With our analysis, we show that given the structures based on various types of ties, the networks need additional conscious coordination efforts. But how can coordination in such an extensive fuzzy network of heterogeneous actors be ensured?

Looking particularly at the National Coordination Authority (NCA) in outbreak management reveals that respondents considered the NCA the “top receiver” and “top spreader” of information. Its position in the collaboration networks is, however, less clear. It is the central actor in the information sharing networks, but what this means for its position as the central coordinator remains a guess. This is mainly due to the fact that we identified several actors (and thus potential coordinators) in the core of those networks around the control measures. We were not able to identify the same actors as brokers in the actor networks. This might be caused by the large amount of interdependencies among the actors. In this study, we did not specifically ask the respondents about their thoughts on (needed) coordination. However, the nominations for collaborations clearly showed us that organizations have expectations towards each other, which could be further explored (e.g., Organization A expecting Organization B to perform a specific task and Organization B indicating that it has no role in this scenario). One possibility is that the NCA spreads the information actors need to perform their tasks and coordinate their work with others. From the information we have here, it is difficult to assess whether we have a “command and control approach,” a “coordination and communication approach,” or a “ network governance approach,” in terms of Moynihan’s work (2009).

The difficulty with positioning any NCA in a response network coping with an infectious disease outbreak might lie in the fact that the same organization needs to fulfill both specific operational expert tasks in the organizational network and network administrative tasks (as defined by Provan & Kenis, 2008). Doing both by the same organization (and partly) or even by the same persons might lead to difficulties. These could be in only acquiring, processing, and evaluating one-sided information, perceived conflicts of interest or shortage of time to exhaustively fulfill both the operational expert as well as the monitoring and coordinating functions when it comes to task division and task allocation. This might lead to even more problems when it comes to contributing to the “integration of effort” (see Puranam, Alexy, & Reitzig, 2014). On the other hand, there are clear reasons for combining expert medical knowledge and coordination authority in an NCA. After all, it is crucial that the central player has immediate access to the best scientific knowledge and information about the outbreak. Weick (2009, Chap. 4) also recognized this dilemma in his analysis of the WNV outbreak in New York City in 1998. Ideally, the NCA is positioned in such a way in the network that it has (1) perceived authority /a mandate to coordinate the actors, (2) sufficient time/resources to pool the available information as impartially as possible, and (3) can recognize, monitor, and assess the evolving organizational network.

Looking at our results, one can argue that given the large size of the Dutch network and the lack of “natural” integration of many actors’ efforts, the NCA can easily become overburdened in its attempts to facilitate all actors. There is a second risk: If the NCA is expected to inform and coordinate all individual actors, this could at least partially be in conflict with its role as an actor coordinating the organizational network.

Given the peculiarities of the evolving organizational network response in a situation of an infectious disease outbreak as well as the findings from our analysis above, another coordination structure to consider could be the core-periphery network structure as recently introduced by Nowell et al. (2018) in their discussion on the structure of effective governance of disaster response networks. Scholars have theorized a core-periphery structure as potentially providing the benefit of both the cohesion and stability of a closed network while also having the flexibility for the network to grow and recognize the importance of new actors who become involved because of the disease outbreak. It might thus be especially relevant given the fact that an organizational network response to an infectious disease outbreak can be expected to consist of many different sub processes, for example integration of scientific knowledge about the pathogen and risk groups with laboratory and epidemiological processes to understand the actual outbreak, developing and supporting the implementation of context specific control measures, (targeted) risk communication to health care professionals and general public , and so forth. The actors involved are organized along other individual and network goals: for example, patient care or water management in the case of the WNV. Consequently, the approach presented can initiate a dialogue among core actors to share information and reflect on the actual situation.

## Limitations and Future Research

Looking at the recent evaluations of national and international outbreaks, we believe that the organizational network governance approach introduced here can strengthen outbreak management. At the same time, we are fully aware that this approach is far from complete and and researchers must address a number of important issues in the future.

In this study, we have in the first place focused on structure as one governance characteristic. This focus has produced important insights, as we have demonstrated in presenting our network analysis for the two scenarios. With this approach, we capture and visualize basic information about the actors as well as about the structure of the collaboration , knowledge, and information exchange. This explorative description creates a basis for practitioners and policy makers to engage in an ex ante assessment and identify and address possible bottlenecks and challenges as laid out above for the two scenarios used here. It also makes it possible to theorize about improving communication and governance of crisis management in general. In this study, we have thus understood “network” primarily as an empirical tool. But insights produced based on an organizational network governance approach used for ex ante and ex post analysis can address questions such as: Which mode(s) of governance and which network structures appear(s) to be most suitable for a rapid response to an outbreak at the (inter)national level? And which methods and data are most appropriate for assessing a response system ex ante or during the event in order to understand the needs and preferences for network-coordination?

However, the data we have collected for this study does not allow us to make any causal statements about the effectiveness of certain structures for the prevention of—or even a response to—an actual event. One way to get a step closer would be to run serious games/simulations with practitioners, in which certain structural characteristics could be manipulated and then assessed by the participants and observers in terms of the effectiveness of the information transmission and coordination. In the long run, studying organizational network responses and their outcomes together with ex ante assessments should improve the field’s understanding of the relationship between structures, governance, and effectiveness of responses under different conditions and for different infectious diseases.

Future research should also unpack the relationship between governance and disasters and complexity in disasters in terms of authority and behavior. Given the absence of features related to legal autonomy and formal authority for the largest part of the organizational network, the question is what role authority plays in these situations and how this can take form. The same is the case for actors’ behavior. What factors actually motivate actors to take on responsibilities in the context of an organizational network to limit the transmission of the virus and its impact, even if public health is not part of their core mission and activity?

Given new global health threats and their potentially enormous impact, it is essential that insights are gained in which governance characteristics of the organizational network produce the type of knowledge necessary for limiting the transmission of the virus and its impact. This is not only vital for coordination in the implementation of measures coping with the outbreak, but already when a situation is perceived and analyzed at the start. As Weick (2009, p. 53) states, “if we spot flaws in collective induction, then we may find an explanation for their genesis in the way people are organizing. Stated more compactly, the degree of intelligence manifest by a network of nodes may be determined by the quality, not just the quantity of its interconnectivity (Taylor and Van Every, 2000:213).”

## Appendix

The results presented in this chapter are based on research that had been conducted since 2015 before the Covid-19 pandemic. Unfortunately, one of the scenario’s that we used for the study, that is, the outbreak of a New Asian Corona Virus, actually came true faster than we could have imagined. The actual outbreak made us aware that our study is based on the implicit assumption that the outbreak of a Corona virus would remain a local outbreak that we would be able to contain. In March, the Dutch public health system was quickly overwhelmed by the speed and the scale of the outbreak that had been happening under the radar since late February in the Netherlands. Therefore, we think that the results are applicable only for the first two weeks in March for the actual outbreak when the system was still in a containment phase. Once the outbreak came into a situation of community spread and was recognized as such, the whole governance system changed significantly. Given the scale and severity of the situation, the response to the outbreak was located at the highest government level on a daily basis and the Dutch prime minister instituted an informal decision and coordination body that met once a week that had not been part of any planning beforehand.

However, even in a changed governance system, we could confirm some of the major findings of the study as well. First, we could see that it is essential that the National Coordination Authority

(1) has a perceived authority/a mandate to coordinate the actors, (2) has sufficient time/resources to pool the available information as impartially as possible, and (3) can recognize, monitor, and assess the evolving organizational network. In addition, we could observe the difficulties we predicted for the coordination of a highly differentiated system of tasks and actors with overlapping activities and roles. The Dutch government reacted after some time mainly by centralizing the coordination of essential tasks such as getting protective material, distribution of ICU beds or testing and tracing. What the actual outbreak also confirmed was the difficult position, the National Coordination Authority finds itself in due to the fact that it fulfills several different roles such as knowledge hub, coordination center, public information organization and the public authority that issues rules and guidelines.

Most importantly, though, we have seen in the actual outbreak that in differentiated democratic societies such as the Netherlands, coping with such outbreaks is not only an epidemiological but also a complex organizational and governance challenge. The perspective we put forward in this paper therefore is very valid and essential and should receive the necessary attention as we move forward in this pandemic and prepare for possible new outbreaks of infectious diseases in the future.
